# Relationships between job stress, post-traumatic stress and musculoskeletal symptoms in firefighters and the role of job burnout and depression mediators: a bayesian network model

**DOI:** 10.1186/s12889-024-17911-5

**Published:** 2024-02-14

**Authors:** Amir Hossein Khoshakhlagh, Saleh Al Sulaie, Saeid Yazdanirad, Robin Marc Orr, Fereydoon Laal

**Affiliations:** 1 Department of Occupational Health, School of Health, Kashan University of Medical Sciences, Kashan, Iran; 2 Department of Mechanical and Industrial Engineering, College of Engineering and Computers in Al-Qunfudah , Umm Al- Qura University, 21955 Makkah, Saudi Arabia; 3 Social Determinants of Health Research Center, Shahrekord University of Medical Sciences, Shahrekord, Iran; 4 School of Health, Shahrekord University of Medical Sciences, Shahrekord, Iran; 5 Tactical Research Unit, Bond University, Gold Coast, Australia; 6 Department of Occupational Health Engineering, Social Determinants of Health Research Center , Birjand University of Medical Sciences, Birjand, Iran

**Keywords:** Job stress, PTSD, Musculoskeletal injury, First responder

## Abstract

**Introduction:**

Job stress, post-traumatic stress disorder (PTSD), and negative psychological outcomes in firefighters can be caused, or aggravated, by their work. These mental disorders can impart musculoskeletal symptoms. This study aimed to investigate relationships between musculoskeletal and psychological disorders in a population of firefighters using a Bayesian network model.

**Methods:**

This cross-sectional study, conducted in 2022, included 2339 firefighters who completed questionnaires during their rest periods. The questionnaires comprised of demographical information, the Occupational Stress Questionnaire-HSE, the PTSD Checklist, Maslach Burnout Inventory, Center for Epidemiologic Studies - Depression scale (CES-D), and Nordic Musculoskeletal Questionnaire. GeNIe academic software was used to analyze the Bayesian network.

**Results:**

High job stress and high PTSD each increased the probability of musculoskeletal symptoms by 34%. When combined, high job stress and high PTSD increased the probability of musculoskeletal symptoms by 37%. Among the mediator’s burnout and depression, depression had the highest association with musculoskeletal symptoms.

**Conclusions:**

Job stress and PTSD can increase musculoskeletal symptoms and are influenced by psychological mediators (like burnout and depression). Adopting preventive and therapeutic measures to mitigate job stress and PTSD, mitigate and rehabilitate WMSD, and manage associated mediators are critical for the mental and physical health of firefighters.

## Introduction

Firefighters are personnel who respond to, suppress the progress of, and extinguish fires in urban and wilderness environments. The main duties of firefighters include responding to fire emergencies, oil spills, vehicle accidents, and various other disasters [1, 2]. These duties can see them exposed to physical dangers such as those imparted by flames, noise, toxic gases, smoke, carbon monoxide, etc., [3, 4]. In addition, firefighters are exposed to unexpected and chaotic environmental stressors (e.g., high temperature environments of over 500 °C), heavy physical loads (e.g., wearing personal protective equipment of 20 + kg and dragging injured people), and psychosocial risks (e.g., concerns over organizational fairness) [5]. Thus, firefighters can face many physical and mental challenges while fulfilling their occupational responsibilities [6, 7].

Musculoskeletal disorders (MSD) refer to any tissue damage to the musculoskeletal system and supporting anatomical elements (e.g., nerves, blood vessels, etc.), and present across a wide range of acute and chronic conditions that can affect muscles, tendons, and ligaments, joints, peripheral nerves, and blood vessels [8, 9]. Clinical presentations can include muscle strains, ligament sprains, tendinopathies (e.g., tenosynovitis, epicondylalgia, etc.,), nerve tractions / compression (e.g., carpal tunnel syndrome, brachial plexus palsy, etc.,) and bone disorders (e.g., stress fractures, osteoarthritis, etc.,) [9–11]. Each injury is unique and is caused by a unique set of circumstances. Various effective risk factors have been recorded in causing these disorders, which include occupational factors such as the work environment, manual work, lifting heavy objects, carrying cargo, and heavy work [9–11].

When the occupational environment and associated duties are considered to contribute to the occurrence of a MSD, the disorder can be termed a work-related musculoskeletal disorder (WMSDs) [9, 11]. In firefighters, for example, muscle strains and ligament sprains, are of particular concern and generally are the most prevalent MSD [1]. Furthermore, these WSMD are often caused by activities that subject the musculoskeletal system to excessive stress [1]. Furthermore, rather than reduce WMSDs, new technologies have been suggested to increase them, through increasing repetitive movements (e.g., typing, scrolling, etc.,) and periods of holding static positions while doing work (e.g., periods in front of screens) and increasing occupational stress and workplace burnout [9, 12, 13]. Given the impacts of WMSD on the community, industry, and the individual, in term of costs and impacts on the individual mitigating WMSD is of importance [1].

One of the main challenges in mitigating WMSDs is the multi-cause nature (including physical, organizational, psycho-social, individual, and cultural-social factors) that contribute to these conditions [14]. For example, the work environment can impart physical demands on the worker that include sustained postures, forceful and repetitive movements, and exposure to vibration [9, 15]. Subsequently, individual risk factors to the sustainment of a WMSD can include age, biological sex, anthropometrics, musculoskeletal and cardiovascular fitness, as well as various psychological factors (e.g., jobs stress, depression, burnout, post-traumatic stress disorder (PTSD), etc.,) [16–18].

Job stress has been described as the adverse reaction of workers when the demands and responsibilities of the workplace are more than the worker can easily manage or are beyond the worker’s ability [18]. Depressive disorders are a common and pervasive problem with serious symptoms and are predicted to become the second cause of disability in humans by the year 5050 [19, 20]. The disorder is also known to significantly reduce worker performance [19, 20]. Job burnout can be considered any mental or physical fatigue caused by dealing with job duties whereby the employee experiences fatigue such that the job demands continuously exceed their energy limits [21]. PTSD is a psychological disorder that may occur after exposure to very threatening events such as accidents, natural disasters, death of loved ones, and other stressful events [22]. The main features of this disorder include intrusive thoughts or memories about traumatic situations, restlessness, anxiety, fear, and increased fight-or-flight reactions. PTSD symptoms can last up to a month but may affect the worker for many years and affect their professional performance [22]. These psychological factors (jobs stress, depression, burnout, and PTSD) are considered adverse health consequences and have been attributed to many physical (MSD) and psycho-social risks observed in the workplace [16, 17, 23, 24].

Given the potential interaction of all these factors a Bayesian network model may assist in determining the relationships between these factors as well as potential mediators. A Bayesian network or “belief network” or “Bayesian network” is a non-circular directed graph that shows a set of random variables and how they are independently related. A Bayesian network can, for example, show relationships between diseases and their symptoms. Therefore, by having symptoms, it should be possible to recognize the possibility of a specific disease in a patient [25, 26]. As such, Bayesian networks are widely used in the field of probabilistic reasoning and become a connected tree based on reasoned probabilities. Bayesian networks become the main subgraph decomposition of the maximum connected tree and are more useful than connected trees. A Bayesian network is exponentially distributed with acceptable initial values and relationships between variables. They are very useful in real world problems especially in occupational health and safety area [25–27].

Given the associations between MSD presentation and mental health, the aim of this study was to investigate relationships between MSD and psychological disorders (depression, job stress, job burnout, and PTSD) in a population of firefighters using a Bayesian network model. This study is the first known investigation of, not only the relationships between MSD, psychological disorders (depression, job stress, job burn out and PTSD) and associated mediating factors in a population of firefighters, but also one using a Bysesian network model in this context.

## Materials and methods

### Participants

The firefighting profession is associated with a high risk of WMSD. In this job, various activities, such as running, climbing, dragging, and lifting, are performed. Moreover, the tasks related to this job are unpredictable and performed in hard environments, which increases the risk level of these disorders. Psychological agents in addition to physical factors can affect musculoskeletal disorders. Some prevalent psychological agents in this job include job stress, PTSD, depression, and burnout. As such, firefighters served as the population of interest. Employing a cross-sectional study design, 2,339 firefighters served as participants.

The participants were selected from 131 fire stations in Northern Iran through random sampling. To initiate the selection process, an inventory was compiled, encompassing 5,199 firefighters from various stations in Northern Iran. Subsequently, a randomized approach was employed to choose 3,000 individuals. Each firefighter was assigned a unique numerical identifier, which facilitated the random selection via specialized software, following which the firefighter’s health records were examined. A total of 2,834 firefighters met the inclusion criteria and were thus extended an invitation to participate in the research project. Outreach was conducted telephonically by the researchers to solicit participation. Of those contacted, 2,617 agreed to participate in the study. Ultimately, 2,339 of the respondents completed the questionnaire, yielding a participation rate of 89%. The inclusion criteria specified an age bracket of 18 to 60 years, a minimum of one year of professional experience, absence of psychiatric disorders, and no current use of psychotropic medication. Conversely, exclusion criteria were designed to omit individuals with a significant trauma history—including vehicular, sports-related, or workplace accidents—rheumatic conditions, previous spinal or major joint surgeries, or musculoskeletal anomalies such as spinal irregularities or genu varum. Additionally, long-term users of corticosteroids or immunosuppressive agents and those unwilling to cooperate throughout the study were precluded. Incomplete survey submissions also resulted in exclusion. The protocol of the study was reviewed and approved by the medical ethics committee of Birjand University of Medical Sciences. All steps of the study were conducted in accordance with the ethical code IR.BUMS.REC.1401.195. All participants consciously completed the consent form provided by the committee.

### Sample size

To calculate the sample size, the Cochran equation was used (Eq. 1). In this formula, *N* = 5199, *p* = 0.5, q = 0.5, z = 1.96, and d = 0.02 were considered. Based on this equation, the sample size was determined as 1643 participants. Given the assumption of participation percentage equal to 80, the minimum sample size was estimated as 2054 participants.

1 $$ n= \frac{N{z}^{2}pq}{N{d}^{2}+{z}^{2}pq} $$

### Data collection

Before data collection, a general description of the study was provided to the participants. Then, the participants answered the questions of the questionnaire during rest periods. All participants were literate. However, the researchers were available to answer any questions and assist participants complete the questionnaires if needed. The questionnaires comprised of demographic information, the Occupational Stress Questionnaire-HSE, the PTSD Checklist, Maslach Burnout Inventory, Center for Epidemiologic Studies - Depression scale (CES-D), and Nordic Musculoskeletal Questionnaire. Completion of these questionnaires took approximately 30 min.

### Tools

#### Demographic information questionnaire

The questionnaire, designed to gather demographic data, encompassed a range of inquiries aimed at creating a descriptive profile of the study participants. This profile comprised of assorted demographic variables such as age, education, marital status, body mass index (BMI), second job status, and smoking status.

#### Occupational stress questionnaire-HSE

The Health and Safety Executive Management Standards Indicator Tool (HSE-MS IT) comprises a structured set of 35 questions crafted to measure psychological factors that are implicated in the assessment of stress-inducing elements at work [28]. This questionnaire consists of seven scales: demands (8 items; a sample item: Different groups at work demand things from me that are hard to combine), control (6 items; a sample item: I can decide when to take a break), officials’ support (5 items; a sample item: I am given supportive feedback on the work I do), colleagues’ support (4 items; a sample item: If work gets difficult, my colleagues will help me), relationships (4 items; a sample item: I am subject to personal harassment in the form of unkind words or behavior), role (5 items; a sample item: I am clear what is expected of me at work), and changes (3 items; a sample item: I have sufficient opportunities to question managers about change at work). Each question is scored on a Likert scale, which ranges from a score of ‘1’ (indicating ‘never’) to ‘5’ (indicating ‘always’), allowing for a total score ranging between 35 and 175. It is important to note that the scoring for certain items is inverted. Within this context, elevated scores on the HSE-MS IT scale are indicative of a diminished likelihood of experiencing occupational stress. The median value was used to divide the occupational stress score into two levels, being low and high. Research conducted by Marcatto et al. [29] affirmed the reliability and validity of the HSE-MS IT in capturing diverse dimensions of job-induced psychological strain.

#### Post traumatic stress disorder inventory

This questionnaire was developed by Weathers et al. in 1994 and was based on the DSM diagnostic criteria for the US National PTSD Center [30]. This instrument which contains 17 distinct items is structured into four primary facets: intrusive cluster (5 items; a sample item: Have you had painful images, memories or thoughts of the events?), avoidance cluster (2 items; a sample item: Have you been avoiding any thoughts or feelings abouts the event?), amnesia and numbing cluster (5 items; a sample item: Have you found yourself unable to recall important parts of the event?), and a hyperarousal cluster (5 items; a sample item: Have you had trouble falling asleep or staying asleep?). Respondents indicate their experiences by selecting from binary choices: ‘yes’ (assigned a value of 1) or ‘no’ (assigned a value of 0). Accumulated scores on this assessment reflect the severity of PTSD symptoms. Higher scores on this scale suggest a higher risk of PTSD. The median value was used to divide the PTSD score into two levels being low and high. Davidson et al. [31] confirmed the reliability and validity of this questionnaire, establishing its strong psychometric properties.

#### Maslach burnout inventory (MBI)

The MBI, conceived by Maslach in 1981, serves as a tool for assessing the burnout [32]. This instrument comprises 22 items that evaluate three core dimensions: emotional exhaustion, which is assessed through nine specific items (a sample item: I feel emotionally exhausted because of my work.), depersonalization, which is assessed through five items (a sample item: I get the feeling that I treat some clients/colleagues impersonally, as if they were objects), and personal accomplishment, which is assessed through eight items (a sample item: I can easily understand how my recipients feel about things in the framework of professional activity). The scoring mechanism of the MBI utilizes a 7-point Likert scale, ranging from a score of ‘0’ (‘never’) to ‘6’ (‘daily’) [33]. To categorize the levels of burnout into low or high, the median score was employed as a threshold. Research conducted by Shamloo et al. [34] has provided evidence supporting the MBI’s validity and reliability, affirming its adequacy for use in gauging burnout.

#### Center for epidemiologic studies depression scale (CES-D)

The CES-D, Developed by Radloff in 1970, serves as an instrument to assess depressive symptoms within a broad population [35]. For the purposes of the research, the comprehensive iteration consisting of 20 items was employed. An illustrative statement from this instrument is “I was bothered by things that usually do not bother me”. Responses to each item are quantified using a 4-point Likert scale, which ranges from score of ‘0’ to ‘3’ (0 = rarely or none of the time, 1 = some or little of the time, 2 = moderately or much of the time, 3 = most or almost all the time), leading to an aggregate score spanning between 0 and 60 with a higher score suggesting a more severe manifestation of depressive symptoms [35]. To categorize the severity of depression among participants, the median score was utilized as a benchmark to delineate low and high levels of depressive symptoms. Konstantinos et al. [36] established that the CES-D’s diagnostic accuracy of this tool was outstanding, surpassing 90%. The reliability of the tool was underscored by a Cronbach’s Alpha coefficient of 0.95, indicating high internal consistency.

#### Nordic musculoskeletal questionnaire (NMQ)

The NMQ, developed by Kuorinka and colleagues in 1987, serves as a diagnostic tool to assess musculoskeletal disorders across various regions including the neck, shoulders, elbows, wrists, back, hips, thighs, knees, and legs [37]. The instrument is bifurcated into two distinct sections: the initial segment gathers demographic data such as work history, weight, and height, and the subsequent segment probes the complication and discomfort in nine body regions over the preceding twelve months. Respondents indicate the presence or absence of discomfort in each specified region with binary responses whereby ‘yes’ (1) indicates affliction and ‘no’ (0) denotes absence. A value of zero was considered as a low level and values of one and greater were considered as high level. Research by Chairani [38] showed that this tool can classify workers who have real pain in the various body regions.

### Data analysis

The statistical tests were carried out using Statistical Product and Service Solutions software (SPSS, IBM, v. 24). Descriptive statistics were calculated following the expectation-maximization method was applied to calculate and replace the missing values. In the study, the interrelationships among different variables were examined through the application of a Bayesian network approach. This method employs a probabilistic graphical framework that delineates the conditional interdependencies among a collection of variables through the utilization of a directed acyclic graph (DAG). This model is used in various sciences, such as ergonomics, for determining association value between variables as well as sensitivity analysis. Bayesian Networks (BNs) were introduced by Pearl [39]. In this study, GeNIe academic software (BayesFusion LLC, v.2.3) was used to model and analyze Bayesian networks. After drawing the Bayesian network graphical structure, a Conditional Probability Table was obtained by the model using the Expectation-Maximization algorithm [40]. A Delta p sensitivity analysis was then applied to rank the parameters [41]. Finally, a 10-fold cross validation analysis was exploited to examine the model validity. Datasets were randomly divided into ten equal folds, nine folds (9 subsamples) were applied to train the Bayesian network model, and the remaining fold (1 subsample) was used to validate the model. A sensitivity analysis also was conducted to examine the effects of the variables [42].

## Results

A total of 2339 firefighters (mean age = 32.30 ± 5.74 years; mean BMI = 26.64 ± 6.39 kg/m^2^) completed the survey. Table 1 summarizes the descriptive statistics related to demographic variables. The majority (55.1%) of the participants were aged 30 to 40 years, with an education degree of associate diploma (37.1%), a body-mass index of 18.5 to 25 kg/m^2^ (42.8%), without second job (55.8%), married (66.9%), and a non-smoker (77.9%).

**Table 1:** Descriptive statistics related to demographic variables (*n* = 2339)

Demographic variables		Frequency	Valid percent (%)
Age (years)	Lower than 30	908	38.8
	30–40	1288	55.1
	41–50	135	5.8
	Higher than 50	8	0.3
Education degree	Under diploma	555	23.7
	Diploma	766	32.7
	Associate degree	867	37.1
	Bachelor degree	149	6.4
	Master degree and higher	2	0.1
Body mass index (BMI)	Lower than 18.5	78	3.3
	18.5–24.9	1001	42.8
	25–30	864	36.9
	Higher than 30	396	16.9
Second job	No	1305	55.8
	Yes	1034	44.2
Marital status	Single	775	33.1
	Married	1564	66.9
Smoking	Yes	516	22.1
	No	1823	77.9

The mean ± SD of the PTSD, job stress, and musculoskeletal symptoms in the participants were 41.54 ± 19.67, 109.50 ± 22.79, and 3.71 ± 2.91, respectively. Moreover, the mean ± SD of emotional exhaustion, depersonalization, personal accomplishment, and depression variables were 22.29 ± 11.82, 11.34 ± 6.37, 26.23 ± 8.18, and 14.43 ± 10.23, respectively. These variables were categorized with the mean ± SD for each group presented in Table 2.

**Table 2:** Studied outcome variables (mean ± standard deviation) (*n* = 2339)

Variable		Frequency	Percent	Mean	SD
PTSD	Low	1659	70.9	30.34	8.95
	High	680	29.1	68.86	9.09
Job stress	Low	1849	70.1	119.13	13.94
	High	490	20.9	73.18	8.68
Emotional exhaustion	Low	1659	70.9	15.59	5.39
	High	680	29.1	38.63	5.77
Depersonalization	Low	1754	75.0	8.10	3.04
	High	585	25.0	21.06	2.93
Personal accomplishment	Low	1501	64.2	31.12	4.90
	High	838	35.8	17.45	4.89
Depression	Low	1759	75.2	9.91	7.06
	High	580	24.8	28.16	4.56
Musculoskeletal symptoms	Low	1185	50.7	0.00	0.00
	High	1154	49.3	5.89	3.25

Table 3 presents the Conditional Probability Table for sleep disorders, which describes the relationship coefficient among the variables.

**Table 3:** The Conditional probability table for musculoskeletal symptoms (*n* = 2339)

PTSD	Job stress	Low	High
Low	Low	0.703	0.297
	High	0.250	0.750
High	Low	0.174	0.826
	High	0.000	1.000

**Fig. 1:**
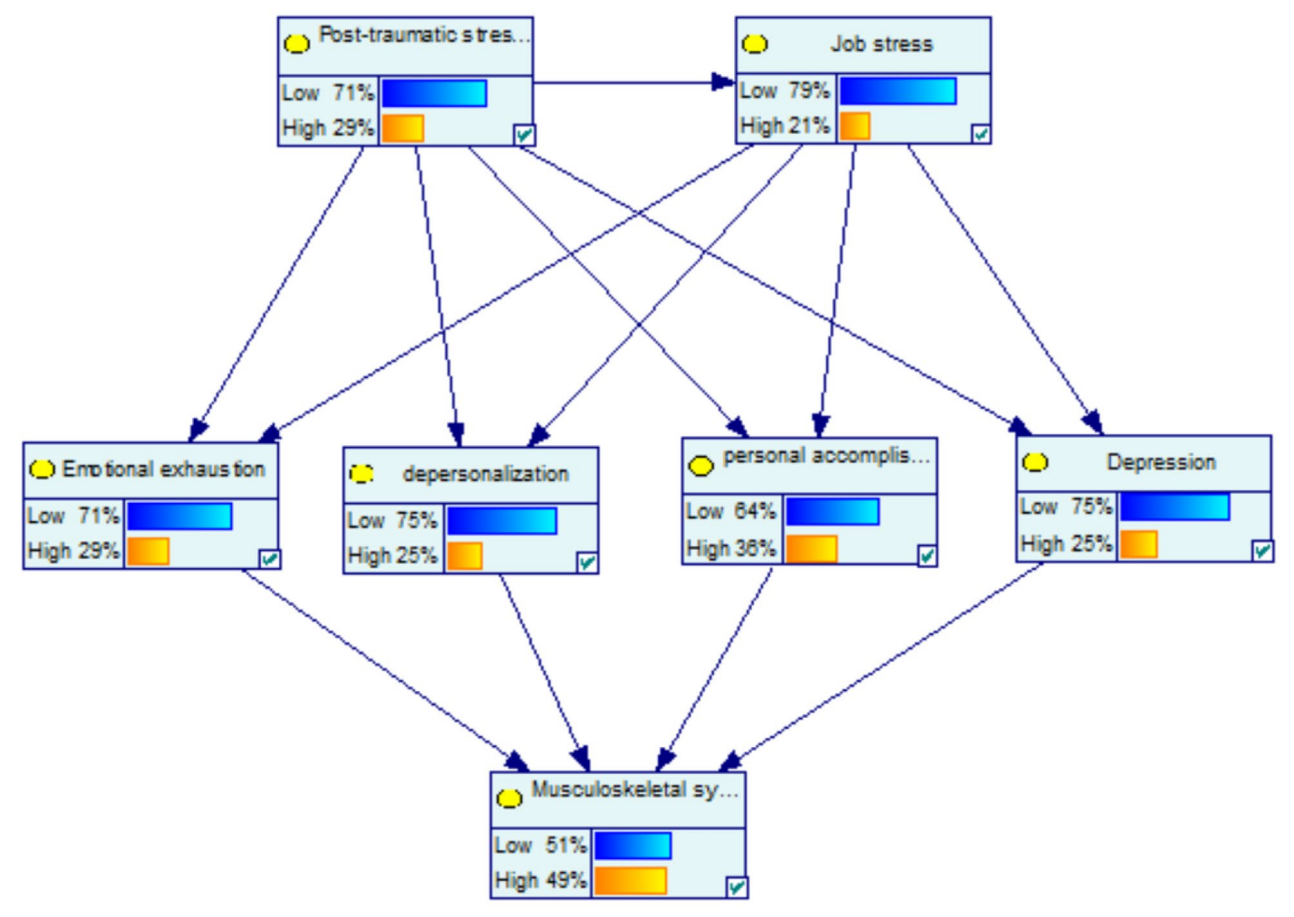
The dependencies among the variables; the marginal probabilities of the studied variables based on the Bayesian network model

**Fig. 2:**
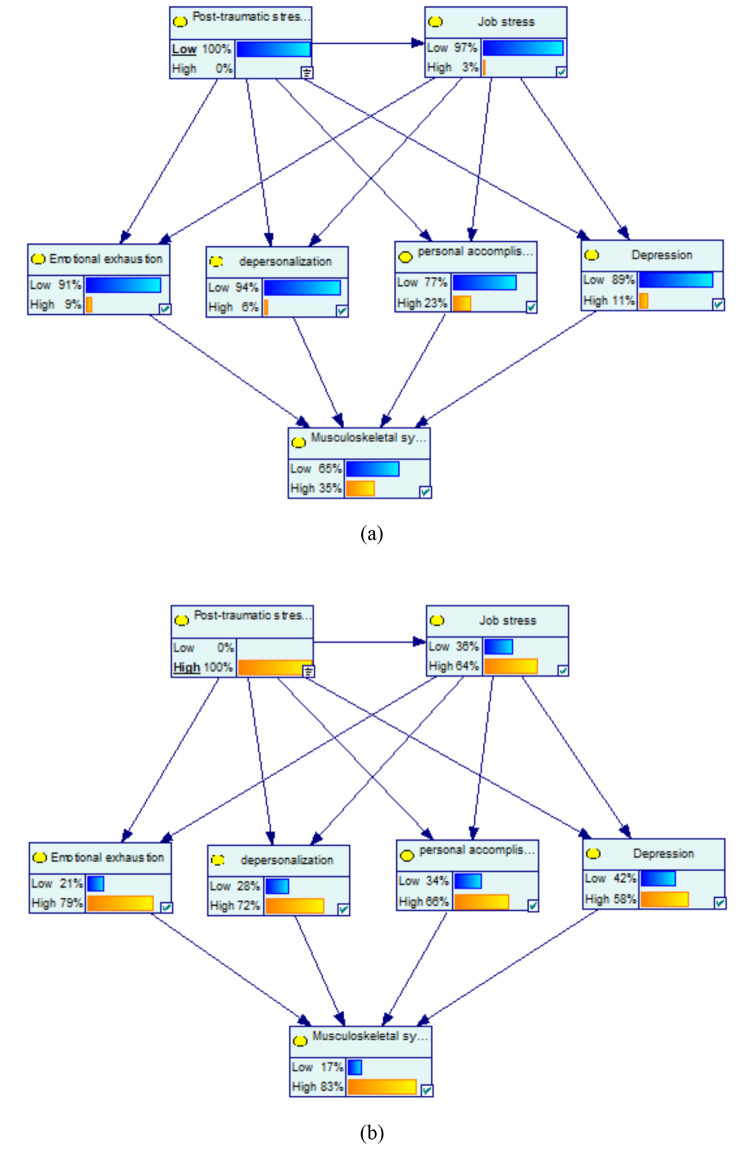
Sensitivity analysis on PTSD: (**a**) low and (**b**) high

Figure 1 shows the dependencies among the variables of the marginal probabilities of the studied variables based on the Bayesian network model. For the low PTSD with the probability of 100%, the probability of the variables of high job stress, high emotional exhaustion, high depersonalization, high personal accomplishment, and high depression decreased by 18%, 20%, 19%, 13%, and 14%, respectively. Among mediator variables, the highest change was related to high emotional exhaustion by -20%. Moreover, at the low PTSD with the probability of 100%, the probability of the variables of high musculoskeletal symptoms decreased by 14% (Fig. 2(a)).

At the PTSD with the probability of 100%, the probability of the variables of high job stress, high emotional exhaustion, high depersonalization, high personal accomplishment, and high depression increased by 43%, 50%, 47%, 30%, and 33%, respectively. Among mediator variables, the highest change was related to high emotional exhaustion by 50%. Moreover, at the high PTSD with the probability of 100%, the probability of the variables of musculoskeletal symptoms increased by 34% (Fig. 2(b)).

**Fig. 3:**
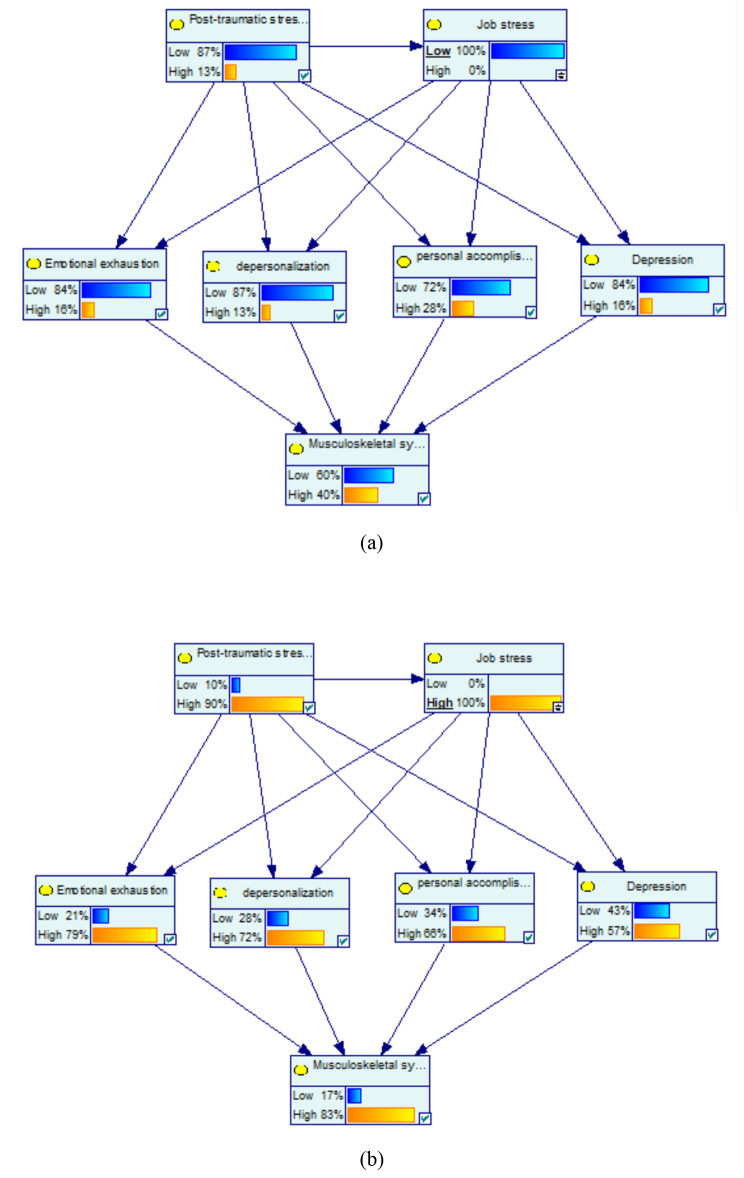
Sensitivity analysis on job stress: (**a**) low and (**b**) high

For the low job stress with the probability of 100%, the probability of the variables of high emotional exhaustion, high depersonalization, high personal accomplishment, and high depression decreased by 13%, 12%, 8%, and 9%, respectively. Among mediator variables, the highest change was related to high emotional exhaustion by -13%. Moreover, at the low job stress with the probability of 100%, the probability of the variables of high musculoskeletal symptoms decreased by 9% (Fig. 3(a)).

At the high job stress with the probability of 100%, the probability of the variables of high emotional exhaustion, high depersonalization, high personal accomplishment, and high depression increased by 50%, 47%, 30%, and 32%, respectively. Among mediator variables, the highest change was related to high emotional exhaustion by 50%. Moreover, at the high job stress with the probability of 100%, the probability of the variables of high musculoskeletal symptoms increased by 34% (Fig. 3(b)).

**Fig. 4:**
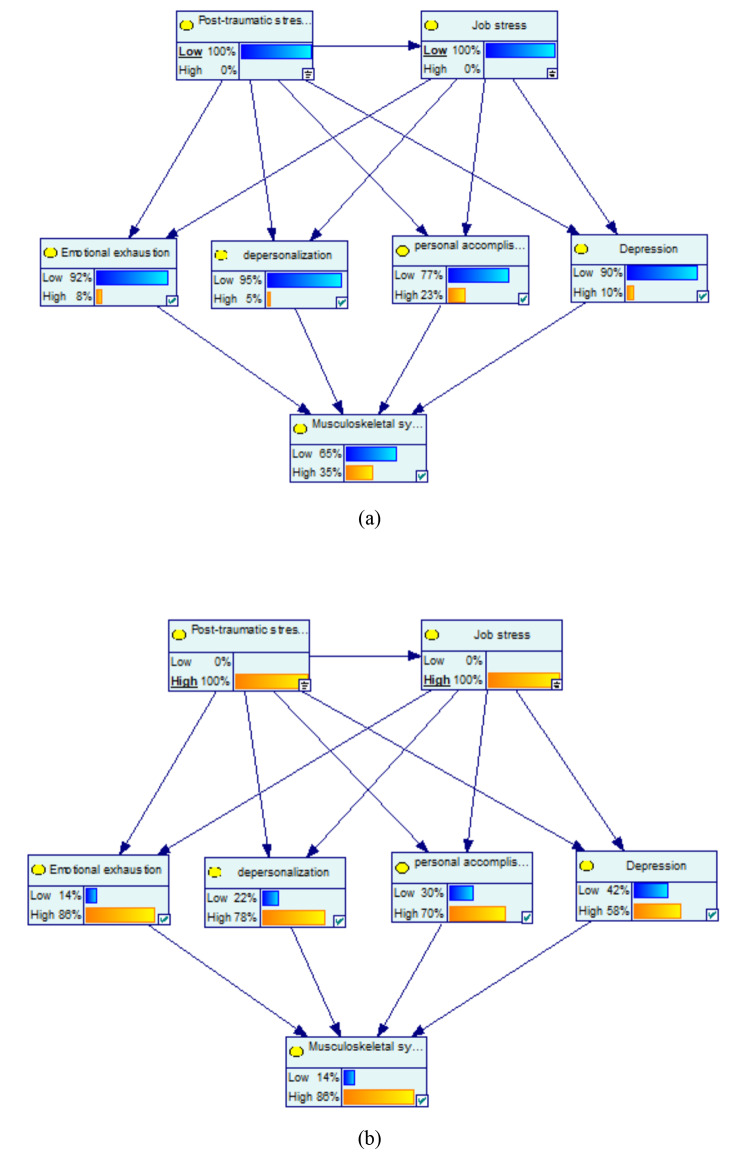
Sensitivity analysis on simultaneously PTSD and job stress: (**a**) low and (**b**) high

Furthermore, at the concurrently low PTSD and low job stress with the probability of 100%, the probability of the variables of high emotional exhaustion, high depersonalization, high personal accomplishment, and high depression decreased by 21%, 20%, 13%, and 15%, respectively. Among mediator variables, the highest change was related to high emotional exhaustion by -21%. Moreover, at the concurrently low PTSD and low job stress with the probability of 100%, the probability of the variables of high musculoskeletal symptoms decreased by 14% (Fig. 4(a)).

Also, at the concurrently high PTSD and high job stress with the probability of 100%, the probability of the variables of high emotional exhaustion, high depersonalization, high personal accomplishment, and high depression increased by 57%, 53%, 34%, and 33%, respectively. Among mediator variables, the highest change was related to high emotional exhaustion by 57%. Moreover, at the concurrently high PTSD and high job stress with the probability of 100%, the probability of the variables of high musculoskeletal symptoms increased by 37% (Fig. 4(b)).

Table 4 represents the computed effective value for the association among the modeled variables. Most effective mean values belonged to the relationship of PTSD and job stress (0.613), relationship of PTSD and emotional exhaustion (0.602), and relationship of PTSD and depersonalization (0.548), respectively. Job stress showed the highest association with depression (0.188) and PTSD had the highest association with job stress (0.613). Also, depression had the highest association with the musculoskeletal symptoms (0.292)

**Table 4:** The computed influence value from the association of the factors in the model

Parent	Child	Influence value	
		Average	Maximum
Depression	Musculoskeletal symptoms	0.292	0.652
Emotional exhaustion	Musculoskeletal symptoms	0.251	0.652
Job stress	Emotional exhaustion	0.174	0.178
Job stress	Depersonalization	0.180	0.182
Job stress	Personal accomplishment	0.094	0.110
Job stress	Depression	0.188	0.362
PTSD	Job stress	0.613	0.613
PTSD	Emotional exhaustion	0.602	0.606
PTSD	Depersonalization	0.548	0.550
PTSD	Personal accomplishment	0.377	0.393
PTSD	Depression	0.304	0.491
Depersonalization	Musculoskeletal symptoms	0.222	0.569
Personal accomplishment	Musculoskeletal symptoms	0.184	0.457

**Fig. 5:**
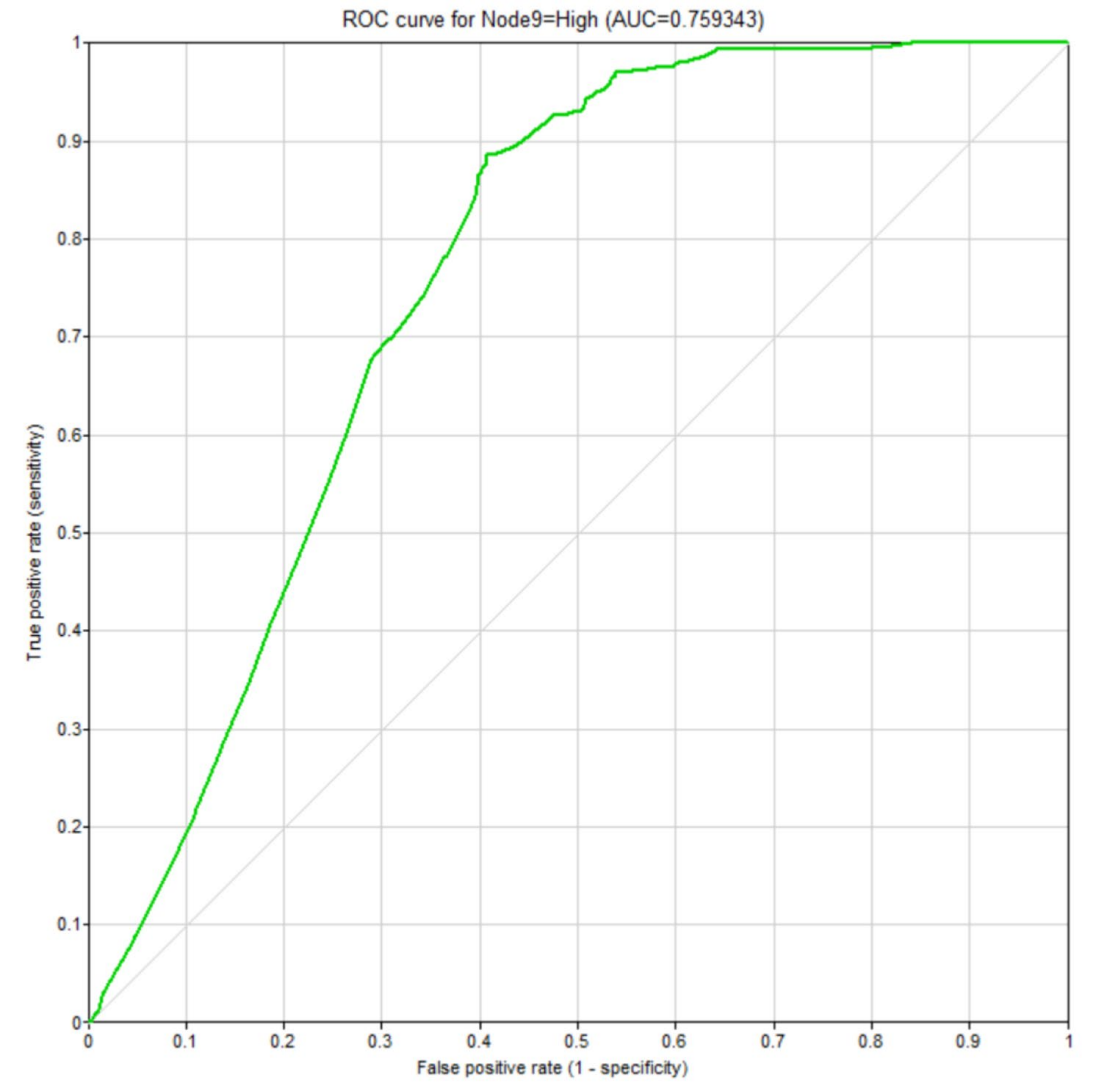
The ROC curve

A Receiver Operating Characteristic (ROC) curve depicted to examine the validity of the fitted Bayesian model is shown as Fig. 5. The area under the curve was equal to 0.759. The confusion matrix related to the classification of the status of the musculoskeletal symptoms was also calculated (Table 5). The values of the sensitivity, specificity, and accuracy of the model were computed as 0.887, 0.593, and 0.742, respectively.

**Table 5:** The confusion matrix related to the classification of the musculoskeletal symptoms

		Predicted	
		Low	High
Actual	Low	684	470
	High	134	1051

## Discussion

This study aimed to investigate relationships between MSD and psychological disorders in a population of firefighters using a Bayesian network model. The modelled results showed that both high job stress and high PTSD each increased the probability of musculoskeletal symptoms by 34%. When combined high job stress and high PTSD increased the probability of musculoskeletal symptoms by 37%. These results demonstrate the relationship between psychological conditions and MSD symptoms in firefighters.

Various sources can create stress in the workplace, such as job demands, decision latitude, social supports, role pressures, job insecurity, and job satisfaction [43]. The results of this study, whereby high job stress was associated with an increased probability of musculoskeletal symptoms is supported by the wider literature. Chakraborty et al. [44] conducted a study investigating relationships between occupational stress and MSDs in Indian construction workers. The results of the study found that occupational stress, as measured using the Occupational Stress Index, had a positive relationship with MSDs (coefficient = 0.357, *p* = 0.001, t = 6.100). Likewise, in a population of Iranian nurses, Barzideh et al. [16] found that the occurrence of MSDs to the back (*p* = 0.002) and lower limbs (*p* < 0.001) were significantly higher in nurses exposed to higher levels of job stress.

Factors leading to job related stress can also manifest in other psychological conditions, such as PTSD. In the study of Japanese firefighters, Saijo et al. [45] reported significant relationships between inter-group conflict (*p* = 0.029), role ambiguity (*p* = 0.010), social support from supervisors (*p* = 0.044), and CES-D scores (*p* = 0.010) and those presenting with PTSD. Won et al. [46] investigated the relationship between PTSD, job stress, and turnover intention in emergency department nurses. The results of their study showed that PTSD can be associated with job stress and turnover intention [46]. As such, the findings of a combined high job stress and high PTSD increasing the probability of musculoskeletal symptoms in this study were therefore not unexpected and is supported by wider research. A systematic review and meta-analysis by Bernal et al., [47], led the authors to postulate a reason for this relationship being that psychosocial items increase perceived stress and thereby impress on physiological responses and subsequently cause musculoskeletal symptoms.

A result of note in this present study was the identification of psychological mediators between job stress, PTSD and MSD. These mediators include burnout dimensions and depression. Abarghouei et al. [48] investigated the relationship between job stress and burnout dimensions in hospital personnel and concluded that there is a significant positive relationship between job stress, emotional exhaustion (*r* = 0.435, *p* = 0.001), and depersonalization (*r* = 0.261, *p* = 0.001), and a negative relationship between job stress and personal accomplishment (*r* = − 0.240, *p* = 0.001) [48]. Li et al. [49] studied the influence of operating room nurses’ job stress on burnout dimensions through path analysis. The results of their study showed that the greatest effect coefficient of job stress on burnout dimensions was assigned to emotional exhaustion (β = 0.750) [49]. While the two aforementioned studies by Abarghouei et al. [48] and Li et al. [49] investigated relationships between job stress and burnout, Mitani et al. [50] evaluated the impact of PTSD and job-related stressors on burnout in fire service workers. The authors found that PTSD was associated with both job stress (*p* = 0.003) and the burnout subscales including emotional exhaustion and depersonalization (*p* < 0.001 respectively) [50].

The results of these studies [48–50] are consistent with the findings of the present study. This result indicates the importance of the role of emotional exhaustion among burnout dimensions and job stress and PTSD. The previous studies also suggest that burnout, particularly emotional exhaustion, is common among emergency occupations [51]. Emotional exhaustion defines as a state of feeling emotionally worn-out and drained as a result of accumulated stress due to the personal or work lives, or a combination of both.

Among mediators in the present study, depression had highest association with musculoskeletal symptoms. The results of a study performed by Choi et al. [52] with health care workers found that depression symptoms (OR = 2.18, 95% CI = 1.07–4.43) (as well as total job stress score (OR = 3.05, 95% CI = 1.62–5.74)) were significantly associated with musculoskeletal symptoms. Likewise, Bekhuis et al. [53] observed that major depressive disorders had strong associations with somatic symptoms, particularly musculoskeletal symptoms (OR = 2.69, 95% CI = 2.18–3.32) [53].

Given that firefighters are constantly at risk of WMSD [1], PTSD [4], and job stress [4], understanding relationships between these variables as well as mediators, like burnout and depression may optimize firefighter health and well-being. For example, job stress can be addressed to prevent WMSD [54], while WMSD should be fully rehabilitated to help mitigate the risk of PTSD [55].

As a point of note, the average BMI of the participants in this study was greater than 25 kg/m^2^ with more than 50% classified as overweight (37%) or obese (17%). These figures are below those found in firefighters from the United Kingdom, whereby 65% were classified as either overweight (54%) or obese (11%) [56] and well below those found in the United States, whereby 86% of firefighters were classified as either overweight (49%) or obese (37%) [57]. While there are associations between BMI and work stress, PTSD, and injury [58], BMI is considered to over-represent firefighters as being overweight or obese due to their generally higher muscle mass [59]. Thus, while BMI may warrant consideration, it should be viewed with caution with further research needed, notably in regards to causality rather than association.

Given the interrelationships between job stress, PTSD and WMSD, several practical applications may serve to mitigate these workplace disorders. Mindfulness programs [60, 61], providing social support [60], increasing and supporting physical activity [62] and occupational recovery strategies (e.g., stress-related conversations, job-related conversations, and hanging out with coworkers and supervisors) [63] all serve as examples of approaches which have had positive impacts on these interrelated factors in firefighters. Further, health lifestyle modification programs, which include increased physical activity, nutritional education, and social support for both activity and dietary behaviors have been found to be effective in firefighters as either a team-centered or individual-oriented intervention [64]. As such, the above recommendations can be applied to groups or individuals, and through interrelationships, would be expected to have a positive impact across multiple workplace disorders.

Several limitations were noted within this study, notably being an exclusively male population, a lack of investigation between the relationships discussed in this study with broader occupational physical factors known to firefighters, and impacts of interventions on job stress, PTSD, and WMSD both in the short term and longitudinally. Given these limitations, future research should include female firefighters given their underrepresented in this profession [65] and potential differences in PTSD and injury presentations (REF). In addition, future research should include consideration of a wider range of occupational physical factors known to firefighters, like physical fatigue from wearing personal protective equipment and dehydration [66]. Finally, in consideration of the practical applications suggested above, intervention studies could be used to determine the future direction of intervention programs and potential longitudinal impacts.

## Conclusions

The results of the present study showed that job stress and PTSD can, separately and in combination, increase musculoskeletal symptoms in firefighters. Furthermore, burnout and depression could mediate the relationship between these factors and musculoskeletal symptoms. The research presented in this study revealed the need to pay attention to the mental health of firefighters as they relate to WMSD prevention and rehabilitation. Given the typically higher rates of WMSD, PTSD, and job stress in this population adopting preventive and therapeutic measures to mitigate impacts and manage associated mediators, like burnout and stress, is critical. Examples could include the proactive treatment of burnout and depression symptoms and management of job stress or the reactive optimized rehabilitation following a WMSD.

## Data Availability

The datasets generated and/or analyzed during the current study are not publicly available because the intellectual property is owned by the funding body. They may be available from the corresponding author on reasonable request containing the approval from the associated funding body.
